# Drought and salinity stresses induced physio-biochemical changes in sugarcane: an overview of tolerance mechanism and mitigating approaches

**DOI:** 10.3389/fpls.2023.1225234

**Published:** 2023-08-11

**Authors:** Rajeev Kumar, Vidya Sagar, Vivek Chandra Verma, Mala Kumari, Ranjit Singh Gujjar, Sanjay K. Goswami, Sudhir Kumar Jha, Himanshu Pandey, Abhishek Kumar Dubey, Sangeeta Srivastava, S. P. Singh, Ashutosh K. Mall, Ashwini Dutt Pathak, Hemlata Singh, Prakash Kumar Jha, P. V. Vara Prasad

**Affiliations:** ^1^ Indian Council of Agricultural Research (ICAR)-Indian Institute of Sugarcane Research, Lucknow, India; ^2^ Indian Council of Agricultural Research (ICAR)-Indian Institute of Vegetable Research, Varanasi, India; ^3^ Department of Biochemistry, Panjab University, Chandigarh, India; ^4^ Integral Institute of Agriculture Science and Technology, Integral University, Lucknow, India; ^5^ Indian Council of Agricultural Research (ICAR)-Indian Institute of Pulses Research, Kanpur, India; ^6^ Indian Council of Agricultural Research (ICAR)-Research Complex for Eastern Region, Patna, India; ^7^ Department of Botany, Plant Physiology & Biochemistry, Dr. Rajendra Prasad Central Agricultural University, Pusa, Samastipur, India; ^8^ Feed the Future Innovation Lab for Collaborative Research on Sustainable Intensification, Kansas State University, Manhattan, KS, United States; ^9^ Department of Agronomy, Kansas State University, Manhattan, KS, United States

**Keywords:** abiotic stress, stomatal conductance, source/sink, antioxidant, photosynthesis, Saccharum species, genome editing, omics

## Abstract

Sugarcane productivity is being hampered globally under changing environmental scenarios like drought and salinity. The highly complex nature of the plant responses against these stresses is determined by a variety of factors such as genotype, developmental phase of the plant, progression rate and stress, intensity, and duration. These factors influence plant responses and can determine whether mitigation approaches associated with acclimation are implemented. In this review, we attempt to summarize the effects of drought and salinity on sugarcane growth, specifically on the plant’s responses at various levels, viz., physiological, biochemical, and metabolic responses, to these stresses. Furthermore, mitigation strategies for dealing with these stresses have been discussed. Despite sugarcane’s complex genomes, conventional breeding approaches can be utilized in conjunction with molecular breeding and omics technologies to develop drought- and salinity-tolerant cultivars. The significant role of plant growth-promoting bacteria in sustaining sugarcane productivity under drought and salinity cannot be overlooked.

## 1 Introduction

Globally, sugarcane (*Saccharum officinarum*) is a commercial crop with high demand. It is extensively used in the sugar industry and the production of bioethanol (Vital et al., 2017). Sugarcane is widely grown in tropical as well as subtropical climatic regions, covers approximately 24.9 million hectares worldwide, and is cultivated in nearly 80 countries with a total production of 174 million tons (OECD/FAO, 2019). Sugarcane productivity and sustainability are both affected by abiotic changes, as are other crops. Climate change increases crop susceptibility to various abiotic stresses (e.g., drought, salinity, temperature, and waterlogging) and biotic stresses (e.g., pests, diseases, and weeds) in current and future scenarios. The severity of these stresses has significant economic and ecological repercussions on sugarcane production systems worldwide. Population growth, which is increasing continuously, could reach approximately 10 billion by 2050 (Food and Agriculture Organization of the United Nations [FAO], 2017), and rapid globalization puts pressure on agricultural land, shifting crop cultivation to marginal and less suitable lands. The adverse effect of environmental changes also leads to yield reductions and total crop production. High and low temperatures, floods, drought, and salinity stresses are major factors that impede crop yield.

Abiotic stresses have harsh impacts on plant morphological, anatomical, and physiological growth. Abiotic stresses, in general, have an impact on stomatal conductance, chlorophyll synthesis, leaf growth, transpiration, photosynthesis machinery, enzyme activity, membrane stability, and ultimately crop productivity (Gujjar et al., 2020). Drought affects nearly 30% of agricultural land globally (Dinh et al., 2017). Drought or water deficit inhibits physiological and biochemical processes such as growth, development, photosynthesis, and respiration, resulting in a loss in total biomass and juice production (Verma et al., 2019; Gujjar et al., 2021). According to the Food and Agriculture Organization of the United Nations (FAO) estimates, drought caused an 80% drop in global productivity (FAOSTAT 2019). Almost one-third (~33%) of the world’s arable land is salinized, which reduces production and yield significantly. In the present scenario, drought (water scarcity) is a major factor leading to lowering cane productivity and sugar recovery. Zhang et al. (2015) observed clear damage in the structure of the chloroplast of sugarcane leaves during drought or water-deficit conditions.

To cope with adverse environmental situations, plants have developed a variety of coping methods, including escape, avoidance, tolerance, or a combination of these. In case of escape, the plant completes its life cycle before the commencement of an unfavorable environment. However, in sugarcane, being a long-duration crop, this mechanism is ineffective. The second mechanism is stress avoidance, which involves developing the mechanism to endure normal growth and development under stress that can be achieved through morphological modification such as reducing leaf width and length, senescence of old leaves, and stomata closure to reduce water loss (Gujjar et al., 2020). The physiological response of plants to cope with stress involves cross-talk of distinct pathways like signal transduction cascades, and phytohormone transduction pathways (Gujjar et al., 2019). As the abscisic acid (ABA) concentration rises, the efflux of K^+^ and Ca^2+^ ions starts, which accumulates in the guard cells. This leads to the closing of stomata and prevents loss of water by upregulation of *soNCED* gene in sugarcane (Li et al., 2013) and several other ABA-responsive genes in plants (Gujjar et al., 2014; Gujjar et al., 2020). Salicylic acid (SA) protects the plant from drought (water deficit) and salinity stresses. SA is a phenolic compound that regulates plant resistance. It binds to the SA receptors and induces SA genes via nitric oxide action. Nitric oxide activates ICS1 gene and the SA pathway (Verma, 2021). Drought stress condition leads to the increased activity of sucrose-phosphate synthase (SPS) over adenosine diphosphate (ADP) glucose pyrophosphorylase in sink tissues, promoting sucrose synthesis over starch by interfering with ADP glucose pyrophosphorylase. Higher sucrose synthesis promotes the biosynthesis of osmoprotectants, which protects plants from osmotic stress.

Genetically engineered plants with compatible solutes such as glycine betaine, sorbitol, mannitol, and proline enhance abiotic stress tolerance (Gujjar et al., 2019; Gupta et al., 2022). Conventional plant breeding can help to cope with abiotic stress by developing tolerant lines of a few selected crops specific for drought, but this technique takes more time and labor, and it is costly (Ashraf, 2010). Breeding techniques like trait-based selection, inheritance studies, marker-assisted selection (MAS), genome-wide association study (GWAS), genetics, and approaches using gene editing and omics (genomics, proteomics, transcriptomics, and metabolomics) are better ways for selecting and developing heat- and drought-tolerant genotypes (Oladosu et al., 2019). The science of stress biology has progressed extensively to develop tolerant plants, and integrating conventional breeding with omics technology will open new avenues for future research. In this review, we have discussed physiological and biochemical changes during drought and salt stresses, and mitigating opportunities using approaches such as genome editing, conventional breeding, and omics technology.

## 2 Physiological responses of sugarcane under drought and salinity stresses

Initial sensing and response communication of plants toward both drought and salinity are nearly identical due to the induction of osmotic stress upon exposure that causes a reduction in growth and stomatal aperture, and nutritional deficiency (like K^+^ and Ca^2+^). However, in addition to dehydration, plants experience ionic stress during prolonged salt exposure, which causes leaf senescence and impairment in photosynthesis leading to a further negative impact on growth (Vasantha et al., 2017). Root elongation increases during long-term drought exposure, as evidenced by the need for plants to access groundwater (Brunner et al., 2015). However, prolonged exposure to salinity stress leads to heavier roots that accumulate more chloride. The accumulation of enormous concentrations of ions, especially Na^2+^, has negative impacts on photosynthetic machinery, resulting in a reduction in enzyme activities as well as the synthesis of pigment. These stressful states reduce the rate of carbon assimilation, while the inability of plants to absorb surplus light leads to enhanced accumulation of reactive oxygen species (ROS), which consequently results in oxidative stress. Most plant species have a mechanism for salt exclusion, which can counteract salt entry into the plant cell by reducing concentration in the cytoplasm via sequestration into the vacuole (Khan et al., 2019). Negative effects on photosynthesis and other physiological activity can be reduced by modifying their physiological processes.

### 2.1 Photosynthesis

**Figure 1:**
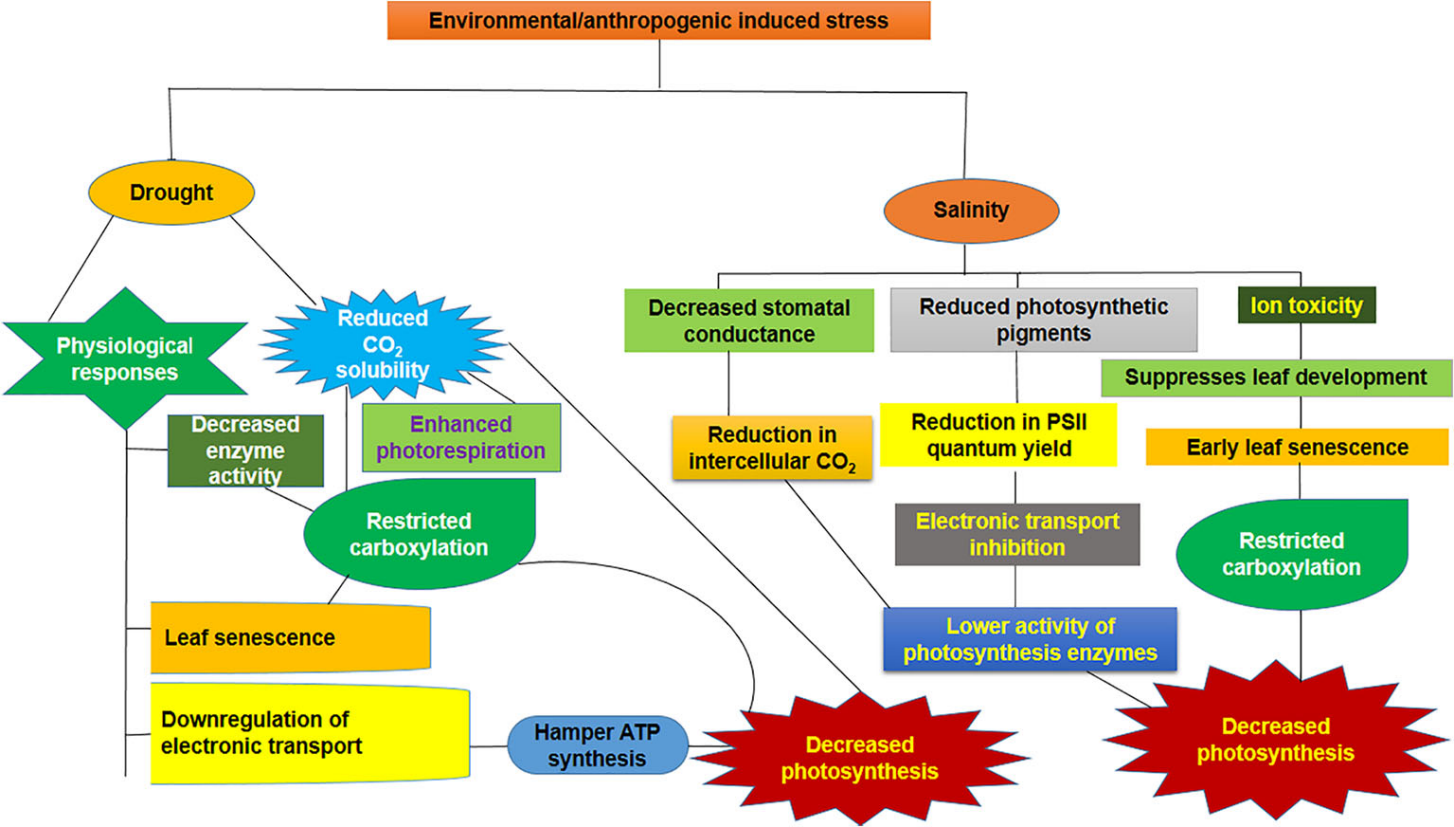
Consequence of drought and salinity on the photosynthetic performance. Downregulation of enzymatic activity as well as electron transport chain (ETC) that leads to rupture of membrane, reduced CO_2_ availability, and senescence of leaf are the events taking place under drought stress. In contrast, salinity leads to ion toxicity, disruption of membrane, reduction in stomatal conductance, lower PSII quantum yield, and slower electron transport rate, which will, in turn, reduce the photosynthetic enzyme activity.

Photosynthesis is a prime physiological process that can be the principal target of drought- and salinity-induced imbalance (Chaves et al., 2009) (**Figure 1**). A high amount of water is required in sugarcane during tillering and the grand growth phase to sustain the physiological activity as well as growth and development, and in such a way, sugarcane is more vulnerable to drought stress at this phase of development (Dinh et al., 2017). Sugarcane, as a C_4_ crop, has a unique CO_2_ concentrating system that gives it an advantage over C_3_ crops in terms of reduction of photorespiration and maximization of its water use efficiency (Ghannoum, 2009; De Souza et al., 2018). Reduced leaf water potential decreased photosynthetic enzyme activity such as ribulose-1,5-bisphosphate carboxylase-oxygenase (RuBisCo), phosphoenolpyruvate carboxylase (PEPC), nicotinamide adenine dinucleotide phosphate-malic enzyme (NADP-ME), and pyruvate, phosphate dikinase (PPDK). The decrease in activity was most pronounced in PPDK (Du et al., 1998). Sugarcane has two distinct modes of C_4_ metabolism depending on their decarboxylating enzymes, namely, NADP-ME and phosphoenolpyruvate carboxykinase (PEPCK), and PEPCK is more prevalent than NADP-ME (Calsa and Figueira, 2007).

A substantial drop in physiological attributes during drought stress in both susceptible and tolerant genotypes of sugarcane can be noticed, while the severity of the adverse effect was pronounced in susceptible genotypes. Higher biomass accumulation, increased photosynthetic rate, and antioxidant enzyme activities were exhibited by tolerant genotypes (Zhang et al., 2020). Physiological processes and the yield of sugarcane were significantly affected under drought stress (Silva et al., 2008; Basnayake et al., 2012). The decline in the photosynthesis rate led to the consequent reduction in the yield during drought (Zargar et al., 2017). Plants can reduce their stomatal aperture in response to drought situations, and when the stress condition persists, impairment in the leaf photochemistry and carbon metabolism results in negative consequences on photosynthesis. Reduced stomatal aperture prevents or minimizes water loss, and this protection system can be considered an adaptive response toward drought onset (Saradadevi et al., 2017).

Drought stress-induced non-stomatal constraint has been described as a major obstacle that inhibits the photosynthesis in sugarcane (Ribeiro et al., 2013), under severe drought, or prolonged moderate drought conditions in sugarcane production systems (Basnayake et al., 2015). Under mild water deficiency, changes in the activity and amount of the RuBisCo were reported at the transcription level (Zhang et al., 2013). Moreover, ROS production in the chloroplast under a stress state reduces the adenosine triphosphate (ATP) production by decreasing the activity of ATP synthase, consequently lowering the regeneration capacity of substrate ribulose-1,5-bisphosphate (RuBP). Early onset of drought stress in sugarcane led to the reduction in the efficiency of photosystem (PS)-II by lowering the maximum quantum yield (Leanasawat et al., 2021). Physiological traits like the maximum quantum yield of PSII efficiency (Fv/Fm), stomatal conductance, and net photosynthetic rate have been investigated in sugarcane during drought stress and recommended for the improvement in yield under drought (Jangpromma et al., 2010; Cha-um et al., 2012).

Even though plants have complex machinery to adapt under osmotic and ionic stresses induced by high salt through an osmotic adjustment with the aid of elevated levels of compatible solute accumulation, drastic retardation of growth at various stages is substantiated due to the highly sensitive nature of sugarcane (Plaut et al., 2000). Reduced photosynthetic efficiency under salt stress conditions debilitates productivity and quality (Akhtar et al., 2003), resulting in a reduction in stalk sucrose concentration (Rozeff, 1995). Reduction in the photosynthetic rate in sugarcane under salinity stress is probably a response against stress in which loss of moisture is prevented through the partial closure of stomata, followed by stomatal conductance reduction, reduced transpiration rate, and, subsequently, limitation in the internal stomatal CO_2_ concentration (Sharma et al., 2021). However, non-stomatal factors such as degradation of chlorophyll and reduction in photosynthetic enzyme activity could also play a major role (Vasantha et al., 2010). Additionally, salt stress influences other components of the photosynthetic machinery, such as chlorophyll and other accessory pigments, biosynthetic enzymes, and carbon fixation competency of the RuBisCo (Demetriou et al., 2007). The susceptibility of light-mediated photosynthetic reactions has been investigated against salt stress and was found to be extremely high. Salinity-induced photosynthesis inhibition is somewhat related to the PSII complex. Reduced PSII activity and a decrease in electron transport quantum yield alter the pigment–protein complex of photosynthesis (Demetriou et al., 2007). Reduction in maximum quantum yield of PSII efficiency (Fv/Fm) in sugarcane under salinity stress due to lesser uptake of water lowers the ATP synthase electrochemical potential as well as photo-system-I, which can further hinder the ATP and NADPH production through interference in photosynthetic apparatus (Silva et al., 2018). Variation in the photosynthetic complex parameters relies on the duration and severity of stress, with plant species being the main factor.

### 2.2 Chlorophyll content

The leaf chlorophyll content of the plant plays a significant role in its photosynthetic capacity. Under salinity or drought conditions, a reduction in chlorophyll content caused by photooxidation of photosynthetic pigments and degradation of chlorophyll is supposed to be a consequence of oxidative stress. Sugarcane is an isohydric plant, capable of maintaining water potential under water-deficit conditions (Meinzer and Grantz, 1990), and this characteristic of the plant helps to use water more efficiently under mild-to-moderate osmotic stress. However, under severe stress conditions, the chloroplast structure of plants is affected due to their chlorophyll content, which leads to a reduction in photosynthetic rate. Different studies have reported that sugarcane chlorophyll concentration decreases in response to osmotic stress (Tang et al., 2021). Sharma et al. (2021) reported a reduction in SPAD (soil plant atmosphere device) reading of sugarcane under salt stress by 56% as compared to their non-stressed counterparts. Under extreme drought stress conditions, the tolerant sugarcane varieties had higher SPAD values (Zhang et al., 2020). Reduction in total chlorophyll content was noticed in both tolerant and susceptible sugarcane clones exposed to salinity, and the level of reduction was less in tolerant clones. Additionally, phenolic and anthocyanin syntheses increased (Wahid and Ghazanfar, 2006). During salt stress, the chlorophyll content of sugarcane becomes reduced, and this impacts the plant’s photosynthetic capacity. Commercial varieties, which have higher chlorophyll content during salt stress, exhibit higher activity of aminolevulinic acid synthase (ALA synthase) or lower chlorophyllase (Dhansu et al., 2022). The interruption in the photosynthetic potential caused by lower photosynthetic pigments reduces the primary production efficiency of the cane crop.

### 2.3 Phytohormonal regulation

The recognition of the stress event initiates cascades of signal transduction; further interaction with the phytohormones transduction of signal via pathway takes place (Harrison, 2012). Moreover, their important role in plant growth endogenous phytohormone assists in the adaptation of plants to drought and salt stresses through manipulation of their molecular and physiological responses (Fahad et al., 2015; Wani et al., 2016). A diverse group of phytohormones such as ABA, cytokinins (CK), SA, indole-3-acetic acid (IAA), jasmonic acid (JA), and gibberellins (GA), biosynthesis, accumulation, and their further distribution are all influenced under drought and salt stress conditions (Eyidogan et al., 2012; Gujjar and Supaibulwatana, 2019). Rodrigues et al. (2011) reported the differential expression of genes associated with hormone metabolism, stress response, signal transduction, and photosynthesis under different levels of drought in sugarcane. Under drought- and salt-induced stress, signal perception triggers the synthesis of phytohormone, abscisic acid, which plays a significant role in the stress response. Stress perceived by plants triggers the ABA synthesis principally in roots. Moreover, synthesis of ABA can also take place in the leaf cells; further, it can be translocated throughout the plant and can regulate the physiological activities such as stomatal aperture, activities of channels, and upregulation of ABA response-associated genes (Gujjar et al., 2014; Fahad et al., 2015; Ullah et al., 2018). Li et al. (2014) suggested that the endogenous synthesis of ABA as well as its exogenous application in sugarcane imparts drought tolerance through enhanced expression of its antioxidative system. A steady decline in the transpiration rate as well as stomatal conductance, associated with increased ABA content, was observed in sugarcane during rising drought conditions introduced for up to 9 days (Li et al., 2016).

Endogenous ABA, as well as exogenous application, regulates the stomatal closing and opening (Wilkinson and Davies, 2002) via one of the various signaling pathways (Neill et al., 2008) involving a network of alternative intermediate molecules such as secondary metabolites and mineral ions (An et al., 2016; Li et al., 2016). ABA-regulated gene in sugarcane, *SoNCED*, upregulated under drought stress, which encodes an enzyme 9-*cis*-epoxy carotenoid dioxygenase, which commits the rate-limiting steps of ABA biosynthesis (Li et al., 2013). Bundle sheath cell’s water status regulation is linked to *SoDip22* (sucrose-phosphate synthase) (Sugiharto et al., 1997). The effect of IAA is extensively accepted for the invigorations of plant growth and their development (e.g., apical dominance, cell elongation, and vascular tissue expansion) (Fahad et al., 2015). This phytohormone appears to carry out growth correspondence during drought and salt stresses (Eyidogan et al., 2012; Iqbal et al., 2014), induce the expression of genes responsible for the initiation of the root meristem, accelerates the root branching, and improves stress tolerance in plant. Li et al. (2016) found an increase in both ABA and IAA contents in sugarcane in response to drought stress.

Salicylic acid, as a phenolic compound, is generally associated with pathogen-mediated protein expression and regulation (Miura and Tada, 2014). Additionally, various reports highlighted the importance of SA in combating drought and salt stresses and defending the plant against them (Fahad and Bano, 2012). Almeida et al. (2013) reported the differential expression of sucrose-phosphate and trehalose-6-phosphate under drought stress in sugar cane following the foliar application of SA.

### 2.4 Source/sink interplay

Carbon assimilation and conversion into the glucose and other sugars are carried out into the source organs (exporter of photosynthates, e.g., completely developed leaves), and photo assimilates are exported to the sink organs (photo accumulation importers, e.g., stems, root, fruits, and seed) for the growth and development of plant organ (Yu et al., 2015). During plant growth phases, communication between organs of source and sink influences the assimilation of carbohydrates and their partitioning/allocation, both of which are closely related to photosynthesis. Reduction in the photosynthetic activity under drought and salt stress conditions exacerbates the effect on carbon flow in sink organs (Courtois et al., 2000; Lebon et al., 2006). Drought and salinity stresses influence phloem loading and sugar metabolism and have been extensively studied (Pattanagul and Thitisaksakul, 2008; Lemoine et al., 2013). Reduced photosynthesis leads to the alteration in carbohydrates at the source and inconsistency in the translocation pattern between source and sink levels under drought and salinity, particularly in sucrose-translocating plants (Pattanagul and Thitisaksakul, 2008). Furthermore, the reduction in demand caused by stress-induced growth limitation raises the sugar concentration.

During drought stress, the expression of various genes including gluconeogenic enzymes fructose-bisphosphate aldolase (Cramer et al., 2007), soluble sugar phosphorylating enzyme hexokinase (Whittaker et al., 2001), and enzyme associated with the biosynthesis of the raffinose family oligosaccharide galactinol synthase (Taji et al., 2002) enhanced, as exhibited by their abundance at the transcript level in the source leaves. Enhancement in the activity of sucrose-phosphate synthase over ADP glucose pyrophosphorylase in sink tissues under drought stress augmented sucrose synthesis over starch by interfering with the ADP glucose pyrophosphorylase (Geigenberger et al., 1997). Maintenance of cell turgidity under drought can be regulated through a higher content of sugar found in the cytosol, which lowers the osmotic potential (Razavi et al., 2011). Subsequently, a decline in photosynthetic activities as well as leaf senescence can be observed. An increase in plant sugar levels under drought stress is probably an attempt by plants to balance their metabolism for the further maintenance of osmotic homeostasis (Giné-Bordonaba and Terry, 2016). Since sugar concentration acts as a differential sensor for the process like, leaf development by the route of senescence may become affected; however, it can be utilized in carbon mobilization and subsequent reallocation to assist the host plant in mitigating drought-induced negative effects (Whittaker et al., 2001).

Irrigation with poor quality water commonly leads to the salinity stress that initially affects the physiological traits of the plants as influenced under drought stress, specifically the early response of the plant, since both the stresses interrupt the water absorption via the root system through osmotic effects (Navarro et al., 2008). Moreover, prolonged exposure to particular stress affects differently, and the transport of sodium into the plant tissues by the route of the transpirational pull leads to sodium toxicity that can be considered as an initial response to stress. The involvement of K^+^ channels in Na^+^ recirculation via leaf phloem to roots led to a decrease in Na^+^ concentration in leaves (Berthomieu, 2003). The distinct mechanisms utilized by plants to alleviate salt stress are either reduction in cytoplasmic salt concentration or curtailed entry of ions into the plant tissues.

## 3 Biochemical responses of sugarcane under drought and salinity stresses

**Figure 2:**
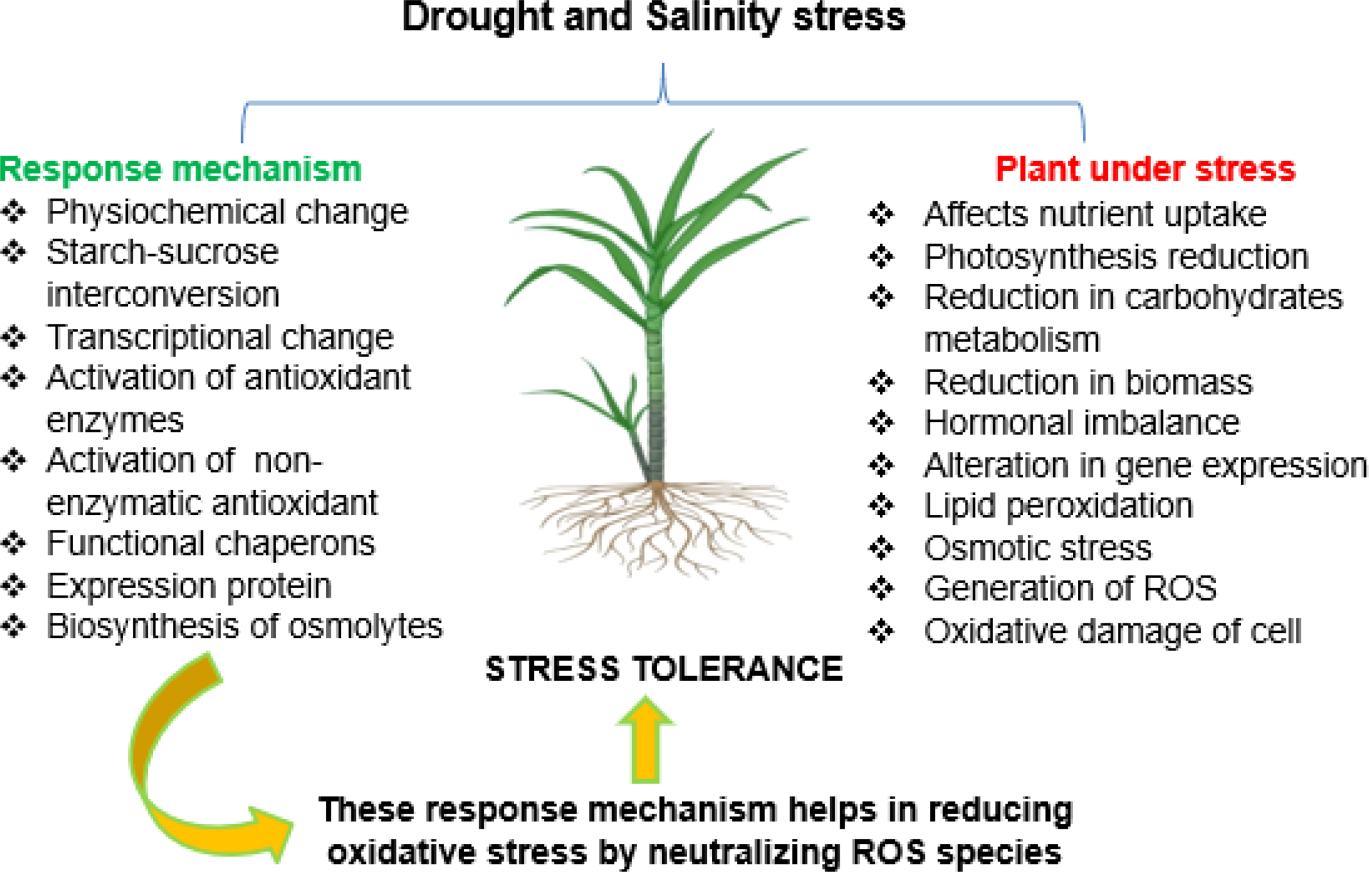
Defense system of sugarcane to combat and tolerate drought- and salinity-generated ROS and their consequence. ROS, reactive oxygen species.

Production of ROS under stress conditions damages membranes and plant biomolecules that affect physiological processes. Antioxidant defense system scavenges the ROS produced in plant cells, which participates in better crop development, plant redox signaling, and plant–microbe mutualism (Hasanuzzaman et al., 2020). Plants utilize enzymatic and non-enzymatic antioxidant defense mechanisms to alleviate the destruction caused by ROS production. Enzymatic antioxidant involves catalase (CAT), superoxide dismutase (SOD), peroxidase (POX), ascorbate peroxidase (APX), lipid peroxidase (LPX), glutathione peroxidase (GPX), and glutathione reductase (GR). The non-enzymatic antioxidants are tocopherols, stilbenes, phenols, ascorbate, glutathione, flavonoids, and carotenoids. In the last decades, flavonoids have been considered a powerful antioxidant in plant systems. These enzymatic and non-enzymatic antioxidant systems and osmolytes quench ROS produced during drought and salinity stresses (Kumar et al., 2021), thereby defending cells from oxidative stress, as shown in **Figure 2**.

The antioxidant defense mechanism has a significant role to prevent drought and salinity stresses and promote crop growth (Gupta et al., 2022). During aerobic metabolism, nearly 1–2% of oxygen uptake by plants is converted into ROS as a by-product in cell organelles like mitochondria, the chloroplast, and peroxisomes. Singlet oxygen, hydroxyl radical, superoxide radical, hydrogen peroxide, alkoxy radicals, and peroxy radicals are classified as reactive oxygen species (Miller et al., 2010; Farnese et al., 2016). Photochemical and electron transport chain reactions mainly contribute to ROS generation in plant cells. Abiotic stress conditions, especially drought and salinity, lead to the exponential hike in ROS production, and antioxidant defense systems are unable to scavenge the ROS produced, leading to oxidative burst that damages biomolecules, osmotic balance, and cellular homeostasis (Maier et al., 2001; Segal and Wilson, 2018).

During the crop growth cycle, plants experience several stresses and utilize their inherent system to combat those stresses, which can be referred to as innate tolerance. However, acquired tolerance is the mechanism that evolved in some plants to overcome these stress conditions. These phenomena acknowledged the immune response of plants against stress (Gupta et al., 2022).

### 3.1 Antioxidant systems

Plants developed complex oxidation–reduction reaction in which ROS are used as markers to regulate normal and stress-related biological processes (Mittler et al., 2011). The activation of the antioxidant defense system is an important chemical process to oxidative stress with respect to plant adaption. Thus, the positive regulation at the transcriptional and post-transcriptional levels of the antioxidant defense system acts as an important marker for stress such as drought and salinity (Gill and Tuteja, 2010). The important ROS scavengers in plants are APX, CAT, and GPX. In comparison to the upregulation of CAT and GPX, the upregulation of APX occurs strongly at the post-transcriptional level. APX is a marker enzyme for the cytosol, chloroplast, and peroxisomes and is found in all plant species. Mittler and Zilinskas (Mittler and Zilinskas, 1994) found higher activity of APX during drought stress in peas. In *Arabidopsis*, *alx8* mutant (altered expression of APX2) has increased tolerance toward drought stress (Estavillo et al., 2011). Transgenic tobacco with overexpressed APX (peroxisomal/cytosolic) from poplar has increased plant performance during drought (Rodrigues et al., 2009). Rodrigues et al. (2009) observed enhanced expression of a peroxidase gene in a drought-tolerant sugarcane cultivar, and a decrease in peroxidase activity is considered to be a limiting step to sugarcane ROS deactivation (Chagas et al., 2008).

CAT is a tetrameric heme-containing enzyme. In peroxisomes, the reaction of catalase involves the dismutation of H_2_O_2_ into H_2_O and O_2_•^−^. An isomeric form of catalase (CAT2) is important during severe drought stress. CAT activation occurs at the post-transcriptional level. The complex regulation of CAT activity involves gene expression, translation, and protein turnover when plants are exposed to severe drought (Sofo et al., 2015). The activities of APX and CAT control redox levels in cells and may contribute to the increased capacity of some sugarcane cultivars to decompose H_2_O_2_ under drought conditions (Jain et al., 2015; Sales et al., 2015). SOD is a class of metalloenzyme. It catalyzes the dismutation of two molecules of O_2_•^−^ into molecular oxygen and H_2_O_2_. The higher activity of SOD isoforms (Mn-SOD, Fe-SOD, Cu, and Zn-SOD) counteracts O_2_•^−^ accumulation in different cell organelles during drought stress. Transgenic plants that are more drought-tolerant expressed higher activity of Cu-Zn SOD (Wu et al., 2016). SOD activity in sugarcane is regulated by water-deficit conditions (Jain et al., 2015). Moreover, drought-tolerant sugarcane cultivars exhibit maximum SOD activity under water deficit (Jangpromma et al., 2012). Different SOD isoforms in sugarcane cultivars with different expression patterns have a major impact on antioxidant response during stress conditions (Jain et al., 2015).

However, low-molecular-weight compounds such as glutathione-*S*-transferase (GST), thiol peroxidases, and GPX engage in an antioxidant defense system. Drought increases the activity of thiol peroxidases such as NADPH-thioredoxin reductase (NTR), ferredoxin-dependent TRX reductase (FTR), and GSH/GRX systems. To overcome the drought stress activity of MDHAR, DHAR, GST, and GR transcripts were also expressed (Vaseghi et al., 2018). PRX plays a major role during redox signaling and information transmission in the cell, which might be their predominant function under drought stress (S.-Z. Zhang et al., 2006; Thalmann and Santelia, 2017). To protect plant cells from oxidative damage, non-enzymatic antioxidant molecules can work in tandem with the enzymatic ROS scavenging system.

### 3.2 Osmolytes

Plants in a drought environment must integrate stress signaling and osmoprotective mechanisms. The biotechnological implication for improving abiotic stress tolerance involves metabolic pathway engineering for a number of osmolytes such as glycine betaine, sorbitol, mannitol, and proline (Gujjar et al., 2018; Gujjar et al., 2019). Transgenic plants engineered with osmoprotectant molecules have improved resistance to drought stress, high salinity, and cold stress (Suprasanna et al., 2016; Gupta et al., 2022). Abiotic stress increases the activity of sucrose catabolic enzymes such as invertase (INV) and sucrose synthase (SuSY). Invertase catalyzes sucrose into glucose and fructose, while sucrose synthase gives uridine diphosphate (UDP)-glucose and fructose. Trehalose is a non-reducing disaccharide sugar that acts as an osmolyte and stabilizes membrane lipids (Suprasanna et al., 2016). Sugars homeostasis is a dynamic process in which sucrose–starch interconversion occurs as per the cellular requirement. Starch metabolism and its associated enzymes perform important roles in alleviating the effects of abiotic stress in plants (Thalmann and Santelia, 2017). In sugarcane, the accumulation of osmolytes in drought conditions prevents damage associated with ROS generation (Guimarães et al., 2008). Proline is an efficient scavenger of ROS. Furthermore, proline can function as a compatible osmolyte and molecular chaperone. During water-deficit conditions, proline is accumulated in plants mainly due to increased synthesis and reduced degradation (Das and Roychoudhury, 2014).

## 4 Breeding for drought and salt tolerance in sugarcane

Among the different methods of sugarcane breeding, the two approaches, i.e., traditional breeding methods (involving parental selection, hybridization, and progeny selection) and molecular breeding methods, that complement the traditional approaches using molecular tools are the major approaches.

### 4.1 Conventional breeding

In the present climatic change scenario, frequent occurrences of abiotic stress such as drought and salt stresses in various regions are major constraints for the sugarcane production system that leads to a decline in tonnage. Despite recent progress in conventional breeding as well as transformation technique, the development of drought- and salt-tolerant sugarcane is still a major challenge. These limitations are due to the complex sugarcane genome, complicated plant responses to water-deficit and salinity conditions, and difficulty in the identification of morpho-physiological traits that could be utilized during the selection processes for the commercial production of drought- and salt-tolerant varieties. The parental selection is very important from the viewpoint of the development of commercial cultivars and depends on the progeny performance (Breaux, 1984). The response of clones to various biotic and abiotic stresses, in addition to cane yield and sucrose content, should be the main criteria for selecting good parental lines under changing climatic conditions. Two types of selection approach, i.e., individual and family selection, are utilized in sugarcane breeding programs. Individual selection approach is used when characters have high heritability, and family selection is used when the heritability of the family mean is higher than that of a single plant (Falconer et al., 1996).

Hybridization techniques include biparental crossing and poly crossing involving two and more than two parents, respectively (Cox, 2000). Hybridization and selection techniques are mainly used in sugarcane breeding programs to generate new recombinant clones with high yield, sugar content, and resistance to stresses. Generally, the parents for breeding for peninsular zones and north Indian plain zone are to be selected on the basis of water regimes, rainfall patterns, and irrigation facilities. As far as yield and sucrose are concerned, the studies suggest that in sugarcane breeding, more importance has to be given to the selection of the female lines than to the male lines (Misra et al., 2020). Drought is a complex trait and involves multiple dynamic interactions. During breeding for drought tolerance, selections for traits such as stalk number, height, diameter, and weight are critical along with the cane weight. Integration of suitable physiological traits in the selection program will be valuable in improving the genotypes against drought and salinity stresses. In the past, traditional breeding methods have been fruitful in the development of drought-tolerant cultivars such as Co-87 and Co-263 (Sreenivasan and Amalraj, 2001). *Saccharum spontaneum*, *Narenga*, and *Erianthus* serve as donor varieties in drought conditions when used as a parent for breeding the variety (Wahid and Ghazanfar, 2006; Patade et al., 2008).

The essential guiding force for adaptive responses to drought and other stresses is genetic variability. Conventional breeding is regulated by Mendelian genetics, which means that traits are passed down from parent to offspring. Plant breeders cross-pollinate parental lines with desired traits to produce progeny with desired characteristics. Conventional breeding has primarily been used to increase genetic variability in sugarcane in order to improve variety. During the grand growth phase, cane formation and elongation occur very actively (Jaiphong et al., 2016), and water stress causes crop production losses of up to 60% (Basnayake et al., 2012). Moderate stress, in contrast, has a positive effect on sucrose yield during the maturity stage. Imposing stress at specific crop stages is an effective method for screening abiotic stress and recording observations on physio-biochemical parameters at different crop intervals. Drought has the greatest impact on complex traits such as shoot, leaf, and root parameters; however, their genetic control varies between genotypes. Under water stress, highly exploitable genetic variation for cane and sugar yield was observed (Hemaprabha et al., 2006; Meena et al., 2013; Basnayake et al., 2015; Meena et al., 2020). Through conventional breeding, varieties that have high yield and high sucrose content and are drought tolerant are produced.

Imposing stress at a specific crop stage is an effective approach for screening abiotic stress and recording the observation of physio-biochemical parameters at various crop intervals. Therefore, screening for these important parameters under the drought stress environment is of paramount importance for drought tolerance breeding. The selection of drought-tolerant genotypes through an indirect selection of physiological traits can also be integrated into the breeding program for the improvement of sugarcane (da Silva et al., 2012; Basnayake et al., 2015). Physiological traits are used for drought-tolerant genotype selection. *S. spontaneum*, *Narenga* spp., and *Erianthus* species are used in the sugarcane breeding program for the incorporation of drought tolerance in sugarcane (Meena et al., 2020). Alternatively, drought tolerance can be increased in the plants through exposure to water stress during the early stage of the life cycle (Marcos et al., 2018; Leanasawat et al., 2021). For the selection of desired clones, clones should first enter zonal evaluation trials after those distinct stages of selection; i.e., ground nursery stage, first clonal stage (C1), second clonal stage (C2), and pre-zonal varietal trial stage (PZVT) are used for selection of desired sugarcane clone. For screening crops for abiotic stresses like drought, waterlogging, and salinity, these stresses are applied at C2 and PZVT stages to identify plants that are high yielding and vigorous and perform better in a particular specific climate.

The screening of drought-tolerant cultivars showing low heritability and high interactions between genotype and environment (G × E) is difficult because of the complexity of traits and genes (Cattivelli et al., 2008). Drought-tolerant lines of some selected crops were produced through conventional plant breeding, but this method is time-consuming, labor-intensive, and costly (Ashraf, 2010). Crop physiology, marker-assisted breeding, GWAS, gene editing, and omics (genomics, transcriptomics, proteomics, metabolomics) are all being used to provide knowledge and tools for plant improvement (Oladosu et al., 2019). Despite the importance of quantitative trait loci (QTLs) and GWAS in identifying genomic regions associated with drought-related traits, genetic variants associated with drought-related traits are largely unknown (Liu and Qin, 2021). Given this, there is an urgent need to integrate modern breeding techniques with multi-omics platforms and high-throughput phenotyping to vastly improve our understanding of drought stress response in sugarcane.

### 4.2 Use of gene pool

**Figure 3:**
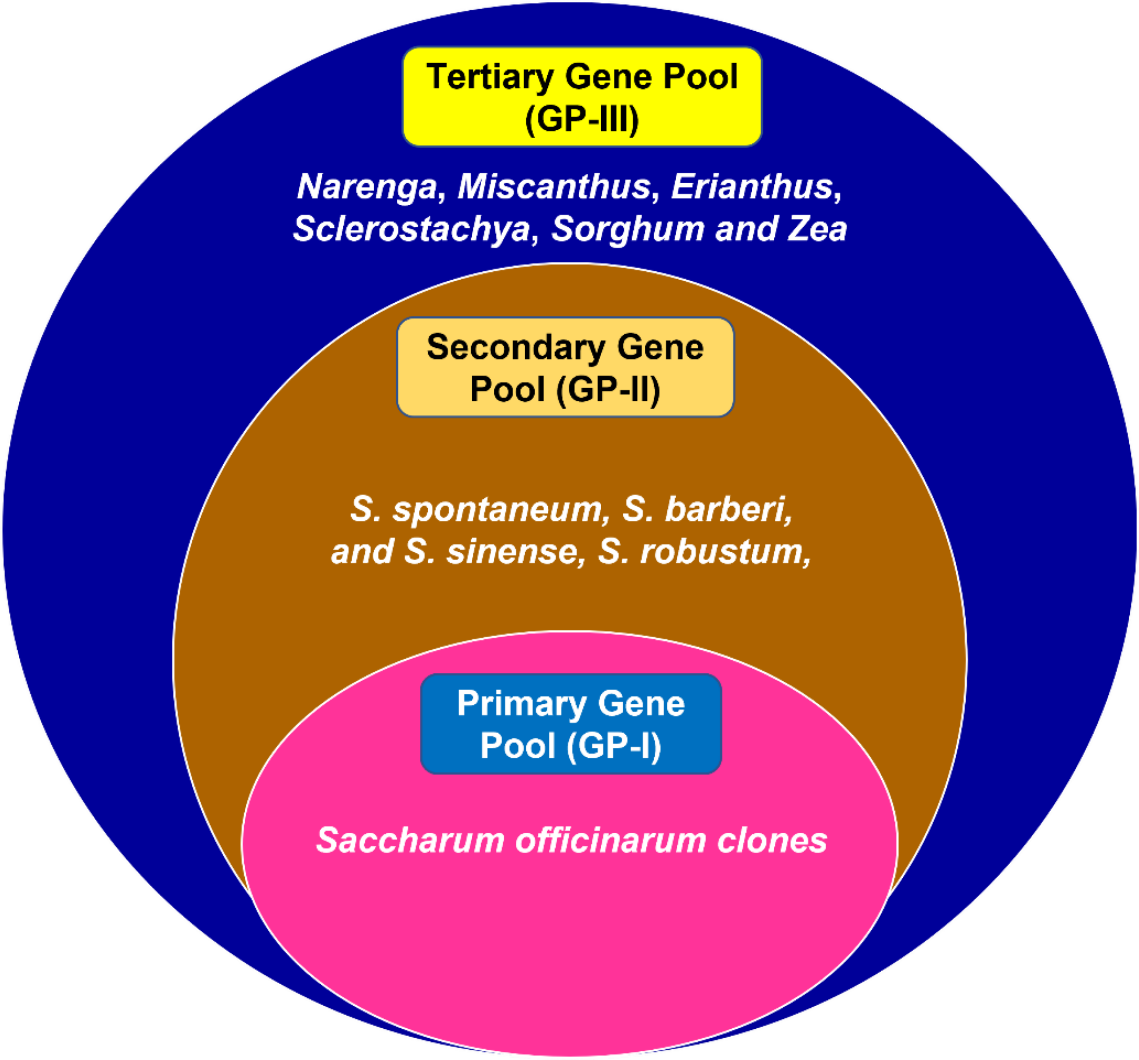
Classification of *Saccharum* species gene pool.

Sugarcane parent species have a complex genome that evolved from a large breeding pool of five closely related genera: *Saccharum* species (*S. officinarum*, *S. spontaneum*, and *Saccharum robustum*), *Erianthus* species, *Miscanthus* species, *Narenga* species, and *Sclerostochya* species (Scortecci et al., 2012). Several *Saccharum* spp. and related genera, including *Erianthus* spp., *Saccharum barberi*, *Saccharum sinense*, and *S. robustum*, are thought to be good sources for instilling salinity tolerance in commercial cultivars (Rao and Sreenivasan, 1985). The ploidy level of *Saccharum* species ranges from 5× to 16× (Manners et al., 2004). Modern sugarcane varieties are produced by interspecific hybridization between *S. officinarum* and *S. spontaneum*. The tropical cane, which is the noble cane *S. officinarum*, has a thicker stem, higher sugar content, and basic chromosome number *x* = 10. *S. spontaneum* is a wild species tolerant to biotic and abiotic stresses with basic chromosome number *x* = 8 (MaChado et al., 2009; Sica, 2021). The varied species of these genera can be classified into various gene pools based on their cross-ability with the cultivated sugarcane. In the primary gene pool (GP-I), the varied species are grouped, which can be easily crossed to produce a fertile hybrid. In the secondary gene pool, the species are crossable with certain difficulties and tend to be sterile. *S. spontaneum*, *S. barberi*, *S. sinense*, and *S. robustum* can be viewed as a secondary gene pool. However, the number of generations of breeding required for the transfer of traits in varietal improvement will be more. In the tertiary gene pool, *Narenga*, *Miscanthus*, *Erianthus*, *Sclerostachya*, *Sorghum*, and *Zea* are included, and the crossing of these species with cultivated sugarcane is difficult to achieve and needs the techniques of embryo rescue and tissue culture (**Figure 3**). The produced hybrids will be weak, lethal, or completely sterile (Sica, 2021).

### 4.3 QTL/candidate gene-based tolerance

Molecular methods are suitable for the development of cultivars for tolerance to one or more traits at once. In sugarcane, the *Agrobacterium*-mediated transformation of *Arabidopsis* tubes pyro-phosphatase (*AVP-1*) was performed to achieve tolerance against drought and salinity stresses (Kumar et al., 2014). New breeding and genomic approaches have been used in recent decades to improve genotypic performance under abiotic stress conditions. Introgression of genes from wild species and related genera for abiotic stress tolerance traits in sugarcane is important in the development of several stress-tolerant varieties. The QTL mapping work has been performed for the disease resistance traits, soluble solid content, and yield traits (Balsalobre et al., 2017); however, to date, there is no report on mapping QTL(s) related to drought and salinity. The sources of genes/QTLs for drought and salinity tolerance need to be explored at a greater pace than the earlier, already reported species of sources such as wild relative *S. spontaneum*, and members of the related genera such as *Narenga* spp. and *Erianthus* spp. (Meena et al., 2020) serve as valuable resources for the same. The utilization of wild and related species in the breeding program is further made difficult due to genome complexity and polyploidy. A successful cross was made to generate an inter-generic hybrid between *Erianthus arundinaceus* and *S. spontaneum* (Lekshmi et al., 2017).

Some of the hybrids have higher root volume and polyphenol component, thereby influencing the extent of drought tolerance (Fukuhara et al., 2013). The transcriptomic analysis of the drought-tolerant and sensitive genotypes led to the identification of several differentially expressed genes (DEGs), and it was noted that the genes such as *MYB*, E3 ubiquitin ligase, small ubiquitin-related modifier-protein (SUMO-protein), *SIZ2*, and aquaporin are drought-responsive genes and transcription factors (Belesini et al., 2017). When salt stress is introduced, an osmotic adjustment mechanism begins to maintain cell turbidity, resulting in slow growth in stressed plants. Changes in morphology, anatomy, water relations, photosynthesis, hormones, ion distribution, and biochemical adaptation may occur depending on genotype, developmental stage, stress intensity, and duration (Liang et al., 2018). Identifying QTLs associated with salt tolerance is an important step toward improving salt-tolerant varieties (Nakhla et al., 2021) and increasing crop production in saline soils. The various QTLs associated with salt tolerance have been identified in cultivated crops such as soybean (*Glycine max* L. Merr.) (Cho et al., 2021), maize (*Zea mays* L.) (Luo et al., 2019), and rice (*Oryza sativa* L.) (Singh et al., 2021), but reports on identified QTL(s) in sugarcane are still lacking. Understanding the magnitude of the impact of salinity on sugarcane crop growth and yield, as well as mapping the salinity stress QTL, should be the primary focus. The list of candidate genes suitable for the improvement of sugarcane for tolerance against salinity and drought is presented in **Table 1**.

**Table 1:** Genes and their function in governing the tolerance against drought and salinity environments.

Trait	Gene	Function	References
Drought and salinity	Trehalose synthase (*TSase*)	Trehalose accumulation	(Zhang et al., 2006)
Drought	Ethylene responsive factor (*SodERF3*)	—	(Trujillo et al., 2009)
Drought	Drought responsive factor (*Scdr 1*)	Signaling cascade	(Begcy et al., 2012)
Drought	Lipoxygenase (*ScLoX*)	—	(Andrade et al., 2014)
Drought and salinity	*Arabidopsis* vacuolar pyrophosphatase (*AVP 1*)	Increasing vacuolar solute content	(Kumar et al., 2014)
Drought and salinity	*Erianthus arundinaceus* DREB2 (*EaDREB2*)	Maintenance of relative water content and photosynthetic rate	(Augustine et al., 2015)
Drought	Sugarcane MYB (*EaMYB18*)	Reduced oxidative damage	(Raza et al., 2016; Saravanan et al., 2018)
Drought	*Arabidopsis* vacuolar H+-pyrophosphatase (*H + PPase*)	—	(Raza et al., 2016)
Drought and salinity	*BcZAT12*	—	(Saravanan et al., 2018)
Drought	*bet A*	Glycine betaine production	(Aloni et al., 2003; dos Santos and de Almeida Silva, 2015)
Drought	Superoxide dismutase (*SOD*)	ROS scavenging	(dos Santos and de Almeida Silva, 2015)
Drought	Indole-3-glycerol phosphate synthase (*IGS*)	Auxin-related gene activity	(Aloni et al., 2003)
Drought	Disulfide isomerase protein (*DEF1*)	—	(Vantini et al., 2015)
Drought and salinity	Late embryogenesis abundance (*LEA*)	Protection of macromolecules	(Sakuma et al., 2006)
Drought	*EaHSP70*	Membrane and protein stabilization	(Augustine et al., 2015)
Drought	Dehydrin proteins (*DHNs*)	Protection of macromolecules	(Iskandar et al., 2011)
Drought and salinity	DEAD-Box Helicase (*PDH45*)	Nucleic acid duplex unwinding	(Augustine et al., 2015)
Drought	Sucrose non-fermenting1-related protein kinase 2 (*SoSnRK2.1*)	Regulation of ROS and antioxidants	(Phan et al., 2016)
Drought	Trehalose synthase gene (*TSase*)	Trehalose accumulation	(S.-Z. Zhang et al., 2006)
Drought	BAX inhibitor (*BI-1*)	Suppressing endoplasmic reticulum stress-induced plant cell death	(Ramiro et al., 2016)
Drought	Cell wall invertases (*ShCWINV*)	Sucrose homeostasis	(Wang et al., 2017)
Drought and salinity	Glyoxalase (*Gly*)	Methylglyoxal metabolism	(Manoj et al., 2019)
Drought	α-Expansin 1 (*EXPA1*)	Plant cell wall modification	(Narayan et al., 2019)
Drought and salinity	Sugarcane drought responsive 2 (*Scdr2*)	Reduced oxidative damage	(Begcy et al., 2019)
Drought and salinity	NAC protein (*SoNAP*)	Senescence-associated function	(Carrillo-Bermejo et al., 2020)
Drought and salinity	Mitogen-activated protein kinase (*ShMAPK*)	Signal transduction pathways	(Ali et al., 2021)
Drought	*Saccharum spontaneum* (*SsDREB*)	Osmotic and photosynthetic regulation	(Li et al., 2021)
Drought	*Dirigent proteins* (*ScDIR*)	Lignin biogenesis	(Li et al., 2022)

## 5 Genome editing approaches

In crops with complicated genomes like sugarcane, introducing a desired feature into a commercial elite variety by traditional breeding is extremely labor-intensive and time-consuming. It takes 12–15 years for a normal breeding method to produce an improved variety (Mohan, 2016; Chen et al., 2017). It is also nearly impossible to introduce several characteristics at a time or to change the metabolic pathways. However, with the introduction of transgenic technology, this was made possible to some extent. With the advent of genome editing (GE) approaches, it may now be performed with significant success and precision. Genome editing is a revolutionary technique in which nucleases create sequence-specific double-strand breaks (DSBs) to insert, remove, or replace DNA at specified locations in the genome of any organism. Non-homologous end joining (NHEJ) or homologous recombination (HR) is used to repair these breaks, resulting in specific mutations. Drought tolerance can be realized via genome editing to target drought-sensitive genes as well as negative regulators of drought tolerance mechanisms in crops. In sugarcane, *ScNsLTP* gene was targeted to modify the response of a novel non-specific lipid transfer protein, thereby catalyzing phospholipid response against the abiotic stress (Chen et al., 2017). There are primarily four different families of genome editing tools or nucleases: mega nucleases, zinc finger nucleases (ZFNs), transcription activator-like effector nuclease (TALEN), and clustered regularly interspaced short palindromic repeats (CRISPR/Cas9).

Zinc finger nucleases were the first endonucleases discovered. These are based on zinc finger proteins, a type of transcription factor found in nature, coupled to the endonuclease FokI. A trinucleotide DNA sequence can be recognized by zinc finger domains. Longer DNA sequences can thus be recognized by a series of connected zinc finger domains, resulting in the necessary on-target specificity. However, zinc finger motifs placed in an array influence the specificity of neighboring zinc fingers, making the design and selection of modified zinc finger arrays more difficult and time-consuming. The final layout’s specificity is difficult to predict because FokI endonuclease is a dimer, and it only cleaves double-strand DNA where two ZFNs attach to opposing DNA strands (Basak et al., 2021). TALENs are bacterial fusion proteins composed of the TALE protein and the FokI endonuclease. Target specificity, like in ZFNs, is derived from the protein–DNA connection (Shan et al., 2013). A single TALE motif identifies one nucleotide in the case of TALENs, while an array of TALEs can interact with a longer sequence. Because each TALE domain’s activity is limited to one nucleotide and does not impact the binding specificity of surrounding TALEs, TALENs are easier to create than ZFNs. TALE motifs are linked to FokI endonuclease, which requires dimerization to cleave DNA, similar to ZFNs. This necessitates the binding of two distinct TALENs in close proximity to the target DNA on opposite strands (Shan et al., 2013). Despite the fact that all three genome editing approaches (ZFN, TALEN, and CRISPR/Cas) are commonly employed, the CRISPR method has few advantages.

Unlike other approaches that rely entirely on protein-based recognition, the CRISPR/Cas system uses fundamental RNA/DNA hybrids to assess sequence specificity. The protospacer adjacent motif (PAM) establishes specificity in the guide RNAs’ (gRNAs’) 20-nucleotide sequence, and the Cas9 enzyme cleaves it. The ability to change multiple genes at the same time, or multiplexing as it is commonly known, is the second significant benefit of this approach, which significantly reduces time. Finally, both ZFNs and TALENs are dimers, and vector creation and plant transformation are difficult processes, whereas CRISPR is simple and efficient. CRISPR/Cas9 technology has emerged as a new tool for editing the sugarcane genome due to its effectiveness and ability to overcome the transgene-silencing problem. This method can easily change several useful genes for many important agronomical traits, even across multiple sites (Kannan et al., 2018). As the sugarcane plant is a glycophyte, drought and salinity stresses have a significant impact on its growth and sucrose content. GE of sugarcane using *Arabidopsis* vacuolar pyrophosphatase (*AVP1*) gene can confer drought and salinity tolerance into sugarcane by the development of a profuse root system. An annotated genome sequence is one of the most important criteria for genome editing because it allows scientists to build specific gRNAs *in silico* that can target specific genes with known or unknown functions (Mohan, 2016).

Drought stress genes and signaling pathways have been better understood due to the advances in genomics analysis techniques like next-generation sequencing (NGS), gene editing systems (Shan et al., 2013), gene silencing (Yin et al., 2014), and overexpression method (Bortesi and Fischer, 2015). These technologies have the advantage of producing small sequence libraries for gRNA design. Sugarcane gRNA design is complicated by the genome’s polyploidy and the lack of a complete genome sequence for commercial variants (Augustine, 2017). To solve this problem, gene expression patterns obtained from RNA-Seq data can be used to assess the sequence variations that exist between different allelic forms. This understanding of the allelic form of sequence diversity can be used to design precise gRNAs that target all allelic forms of the gene of interest. The allele-defined genome of *S. spontaneum* is also available for use as a reference genome. Dimeric RNA-Guided FokI Nucleases (RFNs) can also be used to increase genome editing frequency while minimizing off-target effects (Bortesi and Fischer, 2015). Off-target cleavage in dimeric RFNs is reduced by accurate spacing, gRNA location, and reducing the possibility of an off-target site appearing more than once in the genome (Bortesi and Fischer, 2015).

However, due to the complicated genome, vast genome size, and extremely polyploid and aneuploid nature, there are still many hurdles that must be overcome before this technique can be fully utilized. With a genome size of approximately 10 GB and 8 to 12 homologous gene copies, sugarcane is a classic example of a complex polyploid crop, which poses various challenges in genome editing (Shan et al., 2013). Transgene silencing at both transcription and post-transcriptional levels is a major bottleneck in sugarcane molecular improvement programs (Birch et al., 2010). To tackle off-target cleaving issues, customized Cas9 variants can be used to increase GE efficiency. Overall, despite the fact that significant obstacles remain, the utilization of various Cas9 variants and other CRISPR-associated nucleases could soon be a strong tool for enabling successful GE in sugarcane and other polyploid crops. Genome editing would inevitably increase researcher’s curiosity in producing new and desirable trait modifications in crops in the future. In a country where there is a complete moratorium on trials and uses of genetically modified crops, this could be a highly effective method to generate new improved cultivars of sugarcane.

## 6 Role of omics in acclimation

Recent development in the field of “omics” techniques, specifically ionomics, transcriptomics, metabolomics, and proteomics, provide insight into the mechanism adopted by plants against drought and salinity and are useful in identifying important genes and QTLs that provide tolerance to drought and salinity. Omics can be defined as a biotechnological approach dealing with genomics, proteomics, transcriptomics, or metabolomics. Various omics technologies have been discussed briefly considering drought and salinity in the case of sugarcane under the following subheadings.

### 6.1 Transcriptomics of sugarcane against drought and salinity

Transcriptomics involves the study of total mRNA synthesized by the genome under specific conditions; therefore, it is important to study transcriptome profiling during different stress conditions. Furthermore, alterations in climatic conditions have posed a major threat to agricultural production, which severely impacts the food requirement of the population. In case of severe abiotic stress, plants have developed various kinds of molecular approaches to combat natural stress conditions, in particular the transcription factors (TFs), to deal with different abiotic stress in sugarcane and other crops that can be studied using advanced transcriptomics technology. The sugarcane plant belongs to the Poaceae family, which produces sucrose and is greatly used in agro-based enterprises in different tropical and subtropical regions. To that end, the sugarcane transcriptome profile under stressed conditions must be investigated.

The NAC (no apical meristem (NAM), ATAF1/2, *Arabidopsis* transcription activation factor 1/2, and cup-shaped cotyledon) proteins are a class of TFs that are useful in plant developmental activities and help in providing resistance to various kinds of stresses in different crops. Recently, Belesini et al. (2017) studied the transcriptomic activity of two different sugarcane varieties, “SP81-3250” and “RB855453”, grown under different drought levels with the aid of Illumina HiScanSQ platforms. They observed the upregulation of several genes such as ascorbate peroxidase, E3 SUMO-protein ligase SIZ2, MYB, key enzymes involved in the flavonoid biosynthesis like coenzyme A ligase, and aquaporins, which are responsible for drought tolerance. Furthermore, Belesini et al. (2017) identified various kinds of receptor-like protein kinases (RLKs) and their elicitation upon onset of drought in drought-sensitive varieties; these RLKs are a major player in drought sensing, *bHLH* TFs (basic helix-loop-helix), and 1-aminocyclopropane-1-carboxylic acid oxidase (ACC oxidase) generated in ethylene biosynthesis and different unknown genes. These TFs play crucial roles in abiotic stress resistance (Guo et al., 2017). However, Pereira-Santana et al. (2017) employed a next-generation sequencing technique to unravel the transcriptome profile of the cultivar Mex 69-290 against osmotic stress in Mexico. They observed that enhancement in the expression of genes is related to transcriptional regulation, oxide reduction, carbohydrate breakdown, flavonoids, and distinct kinds of secondary metabolites in the tolerant cultivar. Additionally, genes related to ABA biosynthesis, water regulation, and heat stress were also upregulated.

### 6.2 Proteomics

Proteomics is an advanced study of the proteome that gives useful information about proteins in plant cells at various stages of growth under specific climatic conditions; therefore, it is necessary to study the sugarcane proteome, which helps in determining the drought-resistance mechanism. Sugiharto et al. (2002) isolated and identified a drought-inducible gene *SoDip22* in a drought-stressed cultivar of sugarcane by using two-dimensional gel electrophoresis (2-DE) and reported that *SoDip22* functions in drought stress adaptation in the cells of bundle sheath, which is where ABA-mediated signaling path is induced. An 18-kDa protein was extracted and purified, and 2-DE methodology was applied to identify that protein that was present in leaves of sugarcane subjected to drought conditions (Jangpromma et al., 2007). Various proteins useful in the photosynthesis process and enzymes associated with anti-oxidative injury were isolated and characterized by 2-DE as well as liquid chromatography–tandem mass spectrometry (LC–MS/MS) (Ngamhui et al., 2012). The overexpression of gene *EaDREB2* (dehydration responsive element-binding 2), which was transferred from *E. arundinaceus*, in combination with pea helicase gene *PDH45* (pea DNA helicase), increased drought and salinity tolerance in transgenic sugarcane (Augustine et al., 2015). To understand the impact of drought on protein profiling two contrasting cultivars of sugarcane, RB 72910 (resistant) and RB 943365 (susceptible) were grown under water-stressed conditions for 30 days. The water deficit-associated proteins were identified by using 2-DE and mass spectrometry. Several types of proteins related to photosynthesis, signaling pathways, and regulation processes were either upregulated or downregulated in RB 72910; alternatively, these proteins were downregulated in RB 943365.

Khueychai et al. (2015) used 2-DE accompanied by LC–MS/MS to characterize different types of proteins that were responsive to drought in two different cultivars: K86-161 (resistant) and B34-164 (susceptible). Their findings demonstrated that gene expression of fructose bisphosphate aldolase, O_2_-liberating enhancer proteins, and SOD increased to a greater extent in various parts of K86-161; alternatively, these proteins were found in lower quantities in B34-164 under drought conditions. Furthermore, Salvato et al. (2019) quantified the protein of drought-stressed sugarcane stalk nuclei using filter-aided sample preparation (FASP) and LC–MS/MS techniques. Their result exhibited that most of the 74 exclusive proteins found in control plants are associated with cell wall metabolism, indicating that cell wall metabolism is negatively regulated under drought. Similarly, 37 TFs that were related to different protein domains, e.g., NAC, C2H2 (Cys2-His2), bZIP (basic leucine zipper), C3H (Cysteine3Histidine), LIM (LIN-11, Isl-1, and MEC-3), Myb-related (myeloblastosis viral oncogene homolog), HSF (heat shock factor), and auxin response factor (ARF), were characterized. These TFs are known to be present in nucleus and are synthesized by plant in response to drought conditions.

Moreover, salinity present in the soil is an important problem that affects the sugarcane growth and development process. Pacheco et al. (2013) used 2-DE and LC-MS to analyze the differentially expressed proteome in sugarcane root against the salinity stress in different cultivars and concluded that most proteins accumulated in response to stress are involved in developmental process, carbohydrate metabolism, ROS pathway, protection of protein, and membrane steadiness in resistant cultivar after 2 h of salinity appearance, whereas their presence in a susceptible cultivar was noted after 72 h of salinity stress.

### 6.3 Ionomics

Ionomics is the study of trace elements and mineral nutrients in plant systems. During salinity stress, ion concentrations in different cells are disturbed, but plants can adapt to drought and salinity through osmotic adjustment. Various approaches have been identified in response to highly saline and drought conditions in crop plants. Physiochemical changes in buds of sugarcane were reported in the canes exposed to salinity stress (Rasheed et al., 2016). Salinity causes overproduction of hydrogen peroxide, high amount of Cl^−^and Na^+^ in sugarcane plant tissue, and decreased amount of K^+^ and Ca^2+^, as well as Ca^2+^:Na^+^ and K^+^:Na^+^ proportions, and is useful in the production of different osmolytes in sugarcane plant cells.

### 6.4 Metabolomics

Metabolomics is an advanced technique used for exclusive profiling of all metabolites present in plant cells; therefore, it has been actively used in describing the mechanism of salinity and drought stresses in sugarcane. In recent reports, salt-tolerant and salt-sensitive sugarcane varieties were grown under salinity and drought conditions to identify the various secondary metabolites involved in salinity tolerance. Accumulation of a high amount of proline and lower Na^+^ in leaves of salt-tolerant variety was noticed (Chiconato et al., 2019). Similarly, enhanced levels of phenolic acid, anthocyanin content, and flavone content were beneficial in providing resistance to drought and salinity conditions in sugarcane plants (Molinari et al., 2007; Ali et al., 2019). Furthermore, in order to investigate the physiological and developmental changes in sugarcane-developing buds, which were subjected to salt stress, Rasheed et al. (2016) undertook an experiment, according to which salinity increases the production of hydrogen peroxide, increases the tissue limit of Cl^−^ and Na^+^, decreases the K^+^ and Ca^2+^, and Ca^2+^:Na^+^ and K^+^:Na^+^ ratios, and increases osmolyte synthesis in growing sugarcane buds. Similarly, Vital et al. (2017) in their metabolomics studies revealed that drought and salinity stresses led to a reduction in sucrose content and an increase in the reducing sugar such as glucose and fructose in different sugarcane cultivars. Sugarcane varieties like RB867515 showed significantly higher glucose content when subjected to drought stress. Xylose and inositol sugars also increased during drought and salinity stresses. Moreover, the level of organic acids increased as compared to the levels of pyruvate and isocitrate, which sharply decreased during drought conditions. Several amino acids, which include tryptophan, phenylalanine, tyrosine, leucine, valine, proline, glutamine, lysine, isoleucine, asparagine, and glycine, accumulated in different cultivars of sugarcane like RB867515 and RB855536.

## 7 Soil–plant–microbe interactions and responses

Drought and salinity result in low soil microbial activity and poor plant growth. Soil microbes play a crucial role in the soil during the decomposition of soil organic matter through oxidation, ammonification, and nitrification. Various studies have revealed that some beneficial microbes like plant growth-promoting bacteria (PGPBs) have a positive impact on plant growth promotion under stress conditions through a series of mechanisms including exopolysaccharide (ESP) production, phytohormone production (like indole-3-acetic acid, cytokinin, abscisic acid, gibberellins, and ethylene), regulation of nutrient exchange, and influencing the biosynthesis process of osmoprotectant compounds (e.g., total soluble sugar (TSS), betaine, trehalose, or proline) (Vargas et al., 2014; Gupta et al., 2021a). The indirect way of promoting plant growth by PGPBs is the production of antibiotics, hydrogen cyanide, siderophores, volatile organic molecules, and ammonia, which suppress plant pathogens. PGPBs also play a significant role by modulating molecular pathways, thus inducing the production of different molecules like late embryogenesis abundant (LEA) proteins, lipochitooligosaccharides (LCOs), nodulation factors (NFs), and regulating microbe-associated molecular patterns (MAMP) as well as activating several salt- and drought-responsive genes. Plants with induced systemic tolerance may be influenced by stress-responsive genes mediated by PGPR. In sugarcane, it has been observed that *Gluconacetobacter diazotrophicus* activates genes related to ABA-dependent signaling (Vargas et al., 2014).

### 7.1 Plant growth-promoting bacteria (PGPB)

Production of different osmolytes by microbes protects the plant from drought stress. Increasing the root–shoot biomass through PGPB-mediated IAA production may significantly contribute to coping with drought stress by the plant. Production of aminocyclopropane-1-carboxylate deaminase (ACCD) by rhizospheric bacteria suppresses the ethylene signaling pathway to negatively regulate root drying under water stress conditions. There is also a report of increased proline content in plant leaves and root after injection with drought-tolerant bacteria, thus achieving better plant growth (Yuwono et al., 2005). PGPBs also increase the availability of some chemicals, which play a role in growth promotion and provide micronutrients to the host plant. Production of ESP plays an important role in protection from desiccation (Vanhaverbeke et al., 2003). Secretion of SA by microbes acts as a signaling molecule under drought stress, which triggers genes that act as heat shock proteins (HSPs), antioxidants, and chaperones and also activates genes synthesizing secondary metabolites (El-Daim et al., 2019). It is reported that microbes increase the metabolites like pyruvic acid (PA), thiamine pyrophosphate, uridine diphosphate, succinic acid, and dihydroxyacetone, which helps in combatting drought (El-Daim et al., 2019).

PGPBs can reduce the effects of salt stress through both direct and indirect mechanisms (M’piga et al., 1997; Gupta et al., 2021b), and it was reported that PGPBs act against different phytopathogens by inducing different defense-related enzymes like POX, chitinase, β-1,3-glucanase (GLU), and phenylalanine ammonia lyase (PAL). Extracellular polymeric substances (EPSs) produced by PGPBs bind with positively charged ions such as Na^+^ and reduce the accessibility of toxic ions (Upadhyay et al., 2011). EPS around roots increases the water potential, providing a physical barrier to toxic ions, and improves plant nutrient uptake by plants (Dodd and Pérez-Alfocea, 2012; Zulfiqar et al., 2020). At higher salt concentrations, a greater influx of Na^+^, Cl^−^, Ca^2+^, Mg^2+^, SO_3_^2−^, or CO_3_^2−^ leads to ion toxicity. PGPBs maintain high K^+^/Na^+^ ion ratios and regulate toxic ion homeostasis. It reduces the accumulation of ions like Na^+^ and Cl^−^ in the leaves, increases ion exclusion by the root system, or modulates the ion transporter expression (Zhang et al., 2008; Ha-Tran et al., 2021). A plasma membrane protein, high-affinity K^+^ transporter (HKT), facilitates Na^+^ ion transport in plants, which prevents the over-accumulation of Na^+^ ion concentration in shoots (Zhang et al., 2008). Host–microbe interactions influence the tissue-specific regulation of some genes like HKT-type genes during salt stress to maintain ion homeostasis.

## 8 Conclusions

Sugarcane is an economically important crop that serves as a source of nutrition and energy. Climate change impacts sugarcane crop yield and productivity. Drought and salinity are the two major constraints of sugarcane production. Drought and salinity affect the morphological traits, physiological properties, and enzyme activities, ultimately reducing crop productivity. The first visible symptoms of these abiotic stresses are morphological changes such as leaf rolling, reduced leaf size and number, altered root growth, and stunted growth. To cope with stress conditions, plants have evolved various mechanisms such as escape, avoidance, tolerance, or a combination of these. The diverse gene pools and several wild relatives of sugarcane have served as donors of the resistant QTLs against drought and salinity, which can be introgressed during the development of new cultivars. New techniques such as genome editing and omics technology are opening up new avenues for research to understand the mechanism of drought and salinity stress tolerance. The role of endophytes, and soil–plant–microbe interaction is also highly important in sugarcane crop management against drought and salinity.

## Author contributions

All authors significantly contributed to this article. RK, VS, VCV, MK, RSG, SKG, HS, SKJ, HP, ADP, SS, and AKM prepared the original draft. RK, PKJ, PVVP, SPS, and AP edited and compiled the manuscript. All authors have read and agreed to the published version of the manuscript.
